# CNS-Derived Blood Exosomes as a Promising Source of Biomarkers: Opportunities and Challenges

**DOI:** 10.3389/fnmol.2020.00038

**Published:** 2020-03-19

**Authors:** Simon Hornung, Suman Dutta, Gal Bitan

**Affiliations:** ^1^Department of Neurology, David Geffen School of Medicine, University of California, Los Angeles, Los Angeles, CA, United States; ^2^Brain Research Institute, University of California, Los Angeles, Los Angeles, CA, United States; ^3^Molecular Biology Institute, University of California, Los Angeles, Los Angeles, CA, United States

**Keywords:** biomarker, exosome, extracellular vesicle (EV), neurodegenerative diseases, Alzheimer' disease, Parkinson's and related diseases, ALS

## Abstract

Eukaryotic cells release different types of extracellular vesicles (EVs) including exosomes, ectosomes, and microvesicles. Exosomes are nanovesicles, 30–200 nm in diameter, that carry cell- and cell-state-specific cargo of proteins, lipids, and nucleic acids, including mRNA and miRNA. Recent studies have shown that central nervous system (CNS)-derived exosomes may carry amyloidogenic proteins and facilitate their cell-to-cell transfer, thus playing a critical role in the progression of neurodegenerative diseases, such as tauopathies and synucleinopathies. CNS-derived exosomes also have been shown to cross the blood-brain-barrier into the bloodstream and therefore have drawn substantial attention as a source of biomarkers for various neurodegenerative diseases as they can be isolated via a minimally invasive blood draw and report on the biochemical status of the CNS. However, although isolating specific brain-cell-derived exosomes from the blood is theoretically simple and the approach has great promise, practical details are of crucial importance and may compromise the reproducibility and utility of this approach, especially when different laboratories use different protocols. In this review we discuss the role of exosomes in neurodegenerative diseases, the usefulness of CNS-derived blood exosomes as a source of biomarkers for these diseases, and practical challenges associated with the methodology of CNS-derived blood exosomes and subsequent biomarker analysis.

## Introduction

**Figure 1:**
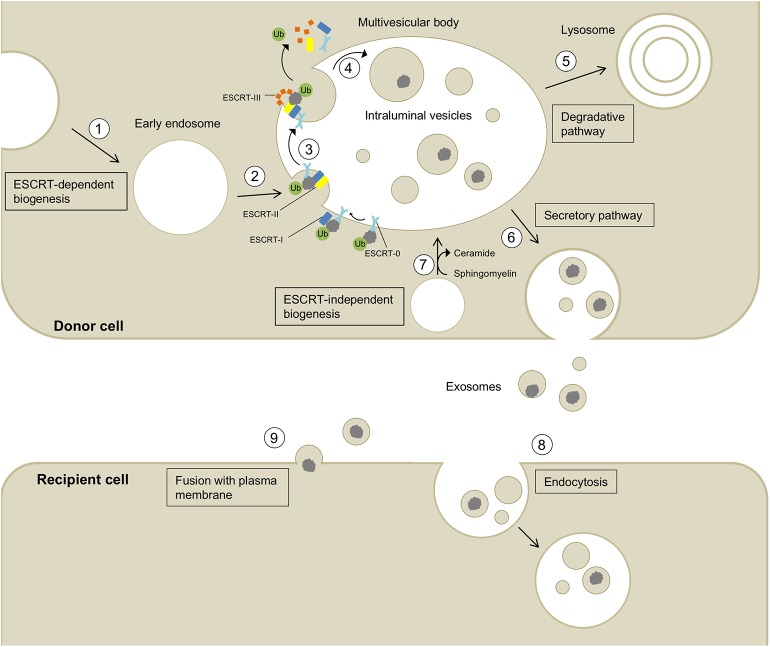
Biogenesis, secretion, and uptake of exosomes and their cargo. (1) Invagination of the cell membrane leads to the formation of early endosomes. (2) ESCRT-0 recognizes and binds ubiquitynated proteins and further recruits ESCRT-I (including the exosomal marker TSG101) and ESCRT-II to this complex. (3) ESCRT-III is a transient protein complex that plugs the inward budding vesicle to avoid the escape of the cargo during scission. Alix recruits deubiquitinases to ESCRT-III, which remove ubiquitin from cargo proteins (Budnik et al., 2016). ESCRT proteins detach from the membrane and are released into the cytoplasm. (4) The formed intralumenal vesicle contains the cargo protein and can either be (5) degraded in lysosomes or (6) secreted into the extracellular space. (7) The formation of intralumenal vesicles can occur via ESCRT-independent pathways and is promoted by higher levels of ceramides in the lipid membrane (Budnik et al., 2016). Exosomes released into the extracellular space can be taken up by recipient cells by endocytosis (8) or fusion with the plasma membrane (9) allowing the transport of cargo between different cells and body parts.

Eukaryotic cells release a variety of extracellular vesicles (EVs), including microvesicles, ectosomes, oncosomes, and exosomes. EVs can be shed directly from the plasma membrane, e.g., ectosomes, or can be released upon fusion of multivesicular bodies (MVBs) with the plasma membrane (Colombo et al., 2014; Coleman and Hill, 2015; Lööv et al., 2016). Exosomes are formed via the latter process by the inward budding of the endosomal membrane, creating MVBs that contain intralumenal vesicles (ILVs). The formation of ILVs is regulated tightly and in many cases depends on endosomal sorting complex required for transport (ESCRT) proteins, and on tetraspanins, including CD9, CD63, and CD81. Alternatively, ILVs can form by ESCRT-independent mechanisms, e.g., by a process mediated by ceramides (van Niel et al., 2011; Perez-Hernandez et al., 2013; Colombo et al., 2014; Thompson et al., 2016). Fusion of MVBs with the plasma membrane leads to the release of ILVs as exosomes, ranging from 30 to 200 nm in diameter (Paulaitis et al., 2018), into the extracellular space where they can be taken up by recipient cells (Figure 1; Coleman and Hill, 2015; Lööv et al., 2016; Thompson et al., 2016). The precise details of the uptake mechanisms of exosomes into recipient cells are not known. In general, exosomes can be taken up by non-specific endocytotic mechanisms, such as macropinocytosis and micropinocytosis, or by more specific, receptor-dependent pathways involving integrins (Hoshino et al., 2015), proteoglycans (Christianson et al., 2013), T cell immunoglobulins, and mucin-domain-containing protein 4 (Tim4) (Miyanishi et al., 2007). Moreover, exosomes can fuse directly with the plasma membrane releasing their cargo into the cytosol of the recipient cell (Figure 1; Montecalvo et al., 2012; Mathieu et al., 2019).

**Figure 2:**
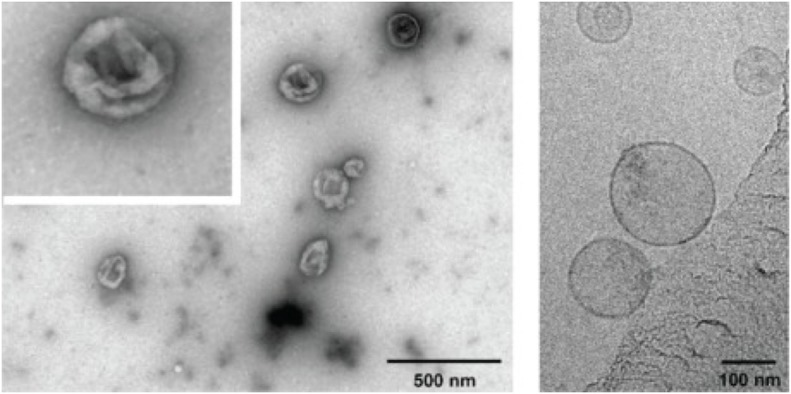
Electron micrographs of exosomes. Exosomes were isolated from cultured primary endothelial cells. Left: exosomes were stained with uranyl acetate and embedded as whole mount preparations in methylcellulose. The image shows a cup-shaped morphology and heterogeneous sizes ranging from 30 to 100 nm. Right: Exosomes were analyzed by cryoelectron microscopy without chemical fixation or contrasting. Exosomes appear as round membranous structures. Adapted from panels B and C in Figure 1 of Banizs et al., © 2014, originally published in International Journal of Nanomedicine (Dovepress). https://doi.org/10.2147/IJN.S64267.

Transmission electron microscopy (TEM) images of negatively stained exosomes initially indicated a cup-shaped morphology, yet later cryo-electron microscopy images of unfixed exosomes, including in a study by Banizs et al., comparing negative-stain EM and unstained cryo-EM of the same exosome preparation, showed a spherical shape, suggesting that the cup-shaped morphology might have resulted from the fixation process in conventional TEM (Figure 2; Théry et al., 2006; Raposo and Stoorvogel, 2013; Banizs et al., 2014).

Exosomes are produced and released by virtually all cell types, including different brain cells, such as neurons, astrocytes, microglia, and oligodendrocytes (Potolicchio et al., 2005; Fauré et al., 2006; Krämer-Albers et al., 2007; Bianco et al., 2009; Dutta et al., 2018; Goetzl et al., 2018; Xia et al., 2019). The nomenclature used in the field is somewhat problematic. Isolation of pure exosomes requires the use of methods such as a sucrose cushion, a density gradient, size-exclusion chromatography, or sequential ultracentrifugation and filtration steps and the resulting vesicles must be characterized thoroughly for their size, morphology, and biochemical characteristics (Lötvall et al., 2014). However, because exosomes are the main type of vesicle in preparations including other types of extracellular vesicles, many authors have used the terms extracellular vesicles and exosomes interchangeably. For simplicity, here we also use the term “exosomes” inclusively when discussing papers that did not go through the rigorous isolation protocols and characterization needed to establish the identity of pure exosome preparation. The reader should keep in mind that in many cases, the preparations described as exosomes may contain also small amounts of other extracellular vesicles.

Originally, exosomes were believed to be a disposal mechanism of unwanted membranes and proteins during the maturation process of reticulocytes into erythrocytes (Pan, 1985). Recent studies have demonstrated that exosomes have multiple additional roles including spreading various proteins, DNA, mRNA, miRNA, and other non-coding RNAs from cell to cell, often depending on the physiological state of the parent cell (Valadi et al., 2007; Guescini et al., 2010; Montecalvo et al., 2012; Cai et al., 2013; Budnik et al., 2016; Sardar Sinha et al., 2018). In the central nervous system (CNS), exosomes play critical physiological roles in intercellular communication, maintenance of myelination, synaptic plasticity, antigen presentation, and trophic support to neurons. Under pathological conditions, especially in proteinopathies, there is an increase in the use of this machinery for disposal of accumulating, unwanted biomolecules (Krämer-Albers et al., 2007; Antonucci et al., 2012; Lee et al., 2012; Budnik et al., 2016; Goetzl et al., 2016; Thompson et al., 2016). In particular, disposal of unwanted cellular components via exosomes occurs to assist when other cellular clearance mechanisms, such as the proteasome and autophagy-lysosome system, gradually fail in eliminating aggregated amyloidogenic proteins (Alvarez-Erviti et al., 2011; Ihara et al., 2012; Urbanelli et al., 2013; Fussi et al., 2018; Miranda and Di Paolo, 2018). Under such conditions, CNS-derived exosomes have been shown to be involved in prion-like, cell-to-cell spread of amyloidogenic proteins, including Aβ, tau, α-synuclein, and TDP-43 (Bellingham et al., 2012; Vingtdeux et al., 2012; Feiler et al., 2015; Polanco et al., 2016).

The role of exosomes in exporting amyloidogenic proteins from brain cells in neurodegenerative proteinopathies has been demonstrated in recent years by multiple groups who showed that CNS-derived exosomes may be enriched in amyloidogenic proteins, such as tau and hyperphosphorylated tau in Alzheimer's disease (AD) and other tauopathies (Clavaguera et al., 2009; Liu et al., 2012; Vella et al., 2016), monomeric and oligomeric amyloid β-protein (Aβ) in AD (Fiandaca et al., 2015; Sardar Sinha et al., 2018), and α-synuclein in Parkinson's disease (PD) (Danzer et al., 2012).

Exosomes have been isolated successfully from human serum and plasma (Caby et al., 2005; Fiandaca et al., 2015), cerebrospinal fluid (CSF) (Vella et al., 2008), saliva (Ogawa et al., 2008; Michael et al., 2010), and urine (Pisitkun et al., 2004). Characterization of exosomes isolated from human serum and plasma has demonstrated similarities in vesicle size, shape, concentration, and presence of exosomal markers suggesting that serum and plasma are equally useful for isolation of blood exosomes (Soares Martins et al., 2018). Multiple exosomal surface markers have been reported and are routinely used to identify exosomes, including the tetraspanins CD9, CD63, and CD81, ALG 2-interacting protein X (ALIX), tumor susceptibility gene 101 protein (TSG101) and ESCRT proteins (Théry et al., 2002; Dutta et al., 2015; Thompson et al., 2016). Exosomes can cross the blood-brain-barrier (BBB) making them a highly attractive source of biomarkers originating in the CNS that could be isolated from the blood (Alvarez-Erviti et al., 2011; Zheng et al., 2017). The ability to cross the BBB in the opposite direction potentially can make exosomes useful for delivering drugs and biomolecules into the brain (Luan et al., 2017; Rashed et al., 2017) though this topic is beyond the scope of our review. Rather, we focus on exosomes that cross the BBB from the CNS into the blood and discuss the role of CNS-derived exosomes in different neurodegenerative disorders, biomarker opportunities, and the technological challenges associated with isolation of CNS-derived blood exosomes.

## Role of CNS Exosomes in Neurodegenerative Disorders

Exosomes have been shown to play a crucial role in the pathology of various neurodegenerative diseases, including AD, PD, amyotrophic lateral sclerosis (ALS), frontotemporal dementia (FTD), and prion diseases (Thompson et al., 2016; Shi et al., 2019). In addition, multiple groups have begun using exosomes as a source of biomarkers for these diseases. The most common fluid biomarkers are listed in Table 1. Some of these biomarkers already have been analyzed in exosomes in one or more diseases, whereas others have been measured in biofluids but not yet in exosomes. This section summarizes briefly the current state of research into the role of exosomes in these diseases whereas the subsequent section discusses their use as a source of biomarkers originating in the CNS.

**Table 1:** List of potential fluid biomarkers for diagnosis of neurodegenerative diseases.

**Disease**	**Biomarker**	**Biofluids**	**References**
AD	Aβ42, pT181-tau, pS396-tau, total-tau	Neuronal exosomes isolated from blood, CSF	Tapiola et al., 2009; Fiandaca et al., 2015; Goetzl et al., 2016; Winston et al., 2016; Jia et al., 2019
PD	α-synuclein, DJ-1	Neuronal exosomes isolated from blood, CSF	Shi et al., 2014; Dutta et al., 2018; Zhao et al., 2019
Prion diseases	PrP, tau, 14-3-3	CSF	Otto et al., 1997; Llorens et al., 2018
FTD	Aβ42, total tau, pT181-tau, pS396-tau, NfL	Neuronal exosomes isolated from blood, CSF	Irwin et al., 2013; Fiandaca et al., 2015; Abu-Rumeileh et al., 2018; Goossens et al., 2018
ALS	TDP-43, NfL, phospho-NfH	CSF, plasma, serum	Kasai et al., 2009; Noto et al., 2011; Boylan et al., 2013; Hosokawa et al., 2014; Lehnert et al., 2014; Lu et al., 2015; Oeckl et al., 2016

*AD, Alzheimer‘s disease; Aβ42, Amyloid β-protein 1-42; CSF, Cerebrospinal fluid; FTD, Frontotemporal dementia; NfH, Neurofilament heavy chain; NfL, Neurofilament light chain; PD, Parkinson‘s disease; PrP, Prion protein; pS396-Tau, Tau phosphorylated at S396; pT181-Tau, Tau phosphorylated at T181; TDP-43, transactive response DNA-binding protein 43 kDa*.

### Alzheimer's Disease

The pathology of AD is characterized by the aggregation of Aβ in senile plaques and of hyperphosphorylated tau in neurofibrillary tangles. Accumulation and spread of the latter lesion in susceptible brain regions correlate with a progressive cognitive decline (Rajendran et al., 2006; Guix et al., 2018). Different roles of exosomes related to both Aβ and tau pathologies in AD have been reported in several studies. Rajendran et al. demonstrated that in HeLa and Neuroblastoma 2a (N2a) cells, sequential cleavage of the amyloid β-protein precursor (APP) by β-secretase occurs intracellularly in early endosomes, where it may be directed subsequently to MVBs and secreted into the extracellular space in exosomes after fusion of the MVB with the plasma membrane. Possibly supporting this idea, the authors showed that the exosomal marker Alix was enriched in the vicinity of senile plaques in the AD brain, whereas the brain of a patient with PD or of a healthy control subject stained negatively for plaques and for Alix (Rajendran et al., 2006).

Other studies have shown that exosomes isolated from neuronal cell cultures accelerated the aggregation of Aβ, suppressed the formation of toxic Aβ oligomers, and facilitated the uptake of Aβ by microglia (Yuyama et al., 2008, 2012). A protective role of exosomes in AD pathogenesis was proposed based on the finding that exosomes isolated from human CSF or brain samples sequestered oligomeric Aβ in the brain (An et al., 2013). The importance of the originating cell type for the physiological effect of exosomes was highlighted by Dinkins et al. who showed that exosomes isolated from astrocytes, in contrast to neuronal exosomes, interfered with the uptake of Aβ in a mixed glial cell culture. However, similar to neuronal exosomes, astrocytic exosomes promoted Aβ aggregation (Yuyama et al., 2012; Dinkins et al., 2014). Recently, it has been found that N2a cells carrying the autosomal-dominant Swedish form of APP (KM670/671NL) secreted exosomes containing Aβ and C-terminal fragments of APP whereas cells expressing wild-type APP secreted exosomes that contained the APP C-terminal fragments but not Aβ. Furthermore, APP and its C-terminal fragments were specifically sorted into exosomes lacking the tetraspanin protein CD63, demonstrating that neuroblastoma cells secrete distinct populations of exosomes containing different cargos and targeting specific cell types (Laulagnier et al., 2018).

Pathologic forms of tau have been shown to spread via exosomes among different cells (Polanco et al., 2016). A study by Clavaguera et al. demonstrated the spreading of the frontotemporal-dementia-associated form P301S-tau from mouse brain extracts to different brain regions after injection into the hippocampus and the overlaying cerebral cortex of transgenic mice expressing wild-type human tau (Clavaguera et al., 2009). The authors did not comment on the potential involvement of exosomes in the spread of tau pathology yet several subsequent publications provided strong evidence for the presence of tau in CNS-derived exosomes and the contribution of such exosomes to the spread of the pathology. Tau and hyperphosphorylated tau have been identified in exosomes isolated from human CSF (Saman et al., 2012; Guix et al., 2018), human plasma and serum (Fiandaca et al., 2015; Guix et al., 2018), brain tissue of transgenic rTg4510 mice (Polanco et al., 2016), and conditioned medium of M1C cells (Saman et al., 2012). Further evidence for a role of exosomes in the transmission of tau has been provided by the findings that microglia secrete and phagocytose exosomal tau and that inhibition of exosome synthesis reduced tau propagation in mouse and cellular model systems (Asai et al., 2015). Exosomes also may contribute to the interplay between Aβ and tau in AD. In a recent study, Aulston et al. demonstrated that treatment with EVs (presumably mostly exosomes), isolated from induced pluripotent stem-cell (iPSc)-derived neuronal cultures generated from a patient harboring the familial-AD-associated A246E of presenilin-1, which increases the Aβ42/Aβ40 ratio, induced tau phosphorylation in wild type C57BL/6 mouse brain (Aulston et al., 2019). Taken together, there is already substantial evidence for the contribution of exosomes to AD pathology, both in spreading the pathology in the brain and potentially in modulating Aβ aggregation. However, this is a developing field and elucidating the precise mechanisms and implications of exosome involvement in AD will require further investigation.

### Parkinson's Disease

The neuropathological hallmark of PD is the accumulation and aggregation of α-synuclein in intracellular inclusions termed Lewy bodies (LBs) and Lewy neurites. α-Synuclein also is the main component of pathological aggregates in related disorders called synucleinopathies, including dementia with Lewy bodies and multiple system atrophy (Spillantini et al., 1998). Several lines of evidence indicate participation of exosomes in the intercellular spread of α-synuclein in the brain. Patients with PD that received transplants of either embryonic nigral neurons (Kordower et al., 2008) or fetal mesencephalic dopaminergic neurons (Li et al., 2008) developed Lewy body-like inclusions in these grafts over a period of 11–16 years, which stained positive for α-synuclein. These finding might be attributed to the prion-like spread of α-synuclein pathology from disease-affected host neurons to the grafts, but also to other factors, such as an unfavorable microenvironment, lack of appropriate trophic signaling, or immune reactions (Kordower et al., 2008; Li et al., 2008). Following up on these initial studies, Hansen et al. explored intercellular α-synuclein transfer in disease propagation using cellular co-culture model systems and transgenic mice. They found that extracellular α-synuclein was taken up by recipient cells via endocytosis and interacted with intracellular α-synuclein. They also demonstrated the *in-vivo* transfer of α-synuclein between host and grafted cells in a mouse model overexpressing human α-synuclein, though this model was not suitable for detecting the potential involvement of exosomes in the transfer (Hansen et al., 2011).

Newly synthesized α-synuclein can be secreted rapidly via unconventional exocytosis and has been found in the lumen of cellular vesicles. Importantly, this intravesicular α-synuclein is more prone to aggregation and is secreted from the cells (Lee, 2005). Proteasomal and mitochondrial dysfunction and other cellular defects associated with PD pathogenesis lead to increased secretion of monomeric and aggregated forms of α-synuclein (Lee, 2005). Emmanouilidou et al. provided the first evidence for exosomal secretion of α-synuclein in a calcium-dependent manner in SH-SY5Y cells. Conditioned medium containing exosomal α-synuclein has been shown to reduce the viability of recipient neurons, suggesting that secretion of α-synuclein contributed to the spreading of PD pathology (Emmanouilidou et al., 2010). Additionally, lysosomal dysfunction is believed to accelerate exosomal α-synuclein release and propagation to surrounding cells (Alvarez-Erviti et al., 2011).

By using a novel protein-fragment-complementation assay, Danzer et al. identified oligomeric α-synuclein species in exosomes in the conditioned medium of human H4 neuroglioma cells and primary cortical neurons. Moreover, they determined that α-synuclein oligomers were present both on the outside and the inside of exosomes, and suggested that α-synuclein could be secreted through different pathways as it was found both free and in association with exosomes (Danzer et al., 2012). In the presence of exosomes, α-synuclein was more prone to aggregation and exosome-associated α-synuclein was taken up more efficiently by cells in culture than free α-synuclein, further supporting a role for exosomes in the intercellular transfer of α-synuclein (Danzer et al., 2012; Grey et al., 2015). A recent study showed that phosphorylated α-synuclein concentration in saliva exosomes was higher in patients with PD than in healthy individuals. The authors also observed a higher abundance of neuronal exosomes in the saliva of patients with PD, which they speculated could reflect increased salivary secretion of exosomes from neuronal endings in salivary glands (Rani et al., 2019).

### Frontotemporal Dementia (FTD) and Amyotrophic Lateral Sclerosis (ALS)

Frontotemporal dementia is a heterogeneous disorder that causes progressive changes in behavior, language, memory, executive control, and motor functions (Olney et al., 2017). It is characterized pathologically by atrophy of the frontal lobe and often involves accumulation of different forms of aberrantly post-translationally modified and aggregated tau in the brain of affected individuals. In addition, FTD can be characterized pathologically by cellular inclusions of the transactive response DNA-binding protein 43 kDa (TDP-43) (Turner et al., 2017), a feature it shares with ALS, which is a distinct neurodegenerative disease affecting motor neurons in the brain and spinal cord. In fact, FTD and ALS appear to be on a spectrum and some patients display mixed phenotypes of both diseases (Kawakami et al., 2019). However, each disease also can present without involvement of the other one and unlike TDP-43, which is shared by both diseases, mutations in certain proteins are associated with either FTD or ALS, but not both. For example, mutations in the superoxide dismutase 1 (SOD1) gene lead to familial forms of ALS but not FTD (Münch et al., 2011). The FTD-ALS clinical spectrum correlates not only with TDP-43 inclusions in neuronal and glial cells, but also with the observation that hexanucleotide-repeat expansion of the C9orf72 gene can lead to ALS, FTD, or a mixed clinical presentation of both diseases (Neumann et al., 2006; Turner et al., 2017).

SOD1 was the first gene discovered to cause familial ALS and the most studied cause of ALS. The presence of SOD1 in exosomes secreted from motor-neuron-like NSC-34 cells overexpressing human wild-type or mutant SOD1 provided the first evidence for the secretion and cell-to-cell transmission of SOD1 in the context of ALS (Gomes et al., 2007). Using a similar cell model, misfolded, mutant, or wild-type human SOD1 was shown to be transmitted between cells both as free protein aggregates and through exosomal transport. In addition, misfolding of human, wild-type SOD1 was propagated in HEK293 cells via exosomes in conditioned media over several passages and was transferred to cultured transgenic mouse primary spinal cord neurons expressing human wild-type SOD1 (Grad et al., 2014). Immunoelectron microscopy using the misfolded-SOD1-specific antibody 3H1 (Pickles et al., 2016; Atlasi et al., 2018) demonstrated that the majority of SOD1 aggregates were present on the exterior of exosomes isolated from the conditioned medium of cultured NSC-34 cells (Grad et al., 2014).

Like SOD1, TDP-43 might be secreted in exosomes, facilitating a prion-like spread of its misfolded species, though to our knowledge, this has not yet been demonstrated directly. Treatment of SH-SY5Y cells expressing TDP-43 with brain extracts of buffer-insoluble proteins from patients with ALS showed that the TDP-43 concentration was increased significantly in exosomes isolated from the conditioned medium compared to untreated cells, whereas the concentration level of the exosomal marker CD63 did not differ between the fractions suggesting that there was no change in exosome concentration (Nonaka et al., 2013). A study by Feiler et al. used a protein-complementation assay allowing the researchers to quantify TDP-43 in HEK-293 cells. Western blot analysis showed the presence of myc-tagged exogenous and endogenous TDP-43 in exosomes of cells transfected with myc-TDP-43. The study showed that exosomal TDP-43 was taken up preferentially by recipient cells and exerted higher toxicity than free TDP-43 (Feiler et al., 2015).

A study by Iguchi et al. provided further support for exosomal transport of TDP-43 using exosomes from the cell-culture medium of N2a cells. Cells expressing mutant forms of human TDP-43 or a fragment thereof secreted the respective protein forms in their exosomes. TDP-43 also was detected in purified exosomes from primary cortical neurons of transgenic C57BL/6 mice expressing human TDP-43^A315T^ but not from primary astrocytes or microglia (Iguchi et al., 2016). The pathological relevance of exosomal TDP-43 was highlighted by the presence of TDP-43 in exosomes isolated from frozen post-mortem temporal cortices of patients who died of sporadic ALS, in which TDP-43 concentration levels were increased compared to exosomes from brains of healthy controls. Treatment of N2a cells overexpressing human TDP-43 with GW4869 or siRNA silencing Rab27A to reduce exosome secretion (Ostrowski et al., 2010) resulted in increased intracellular levels of insoluble TDP-43 aggregates (Iguchi et al., 2016). The studies presented above suggest that TDP-43 is secreted via exosomes in the human brain and that the exosomes are involved in the spread of TDP-43 pathology, common to ALS and FTD, yet a conclusive demonstration of these processes is still not available.

### Prion Diseases

Transmissible prion encephalopathies, such as Creutzfeldt-Jakob disease, scrapie, and bovine spongiform encephalopathy are characterized by misfolding of the normal prion protein PrP^c^ into the aggregation-prone form PrP^Sc^ (Prusiner, 1982, 1991). The first suggestion of an association of misfolded prion protein with exosomes came from a ME7 scrapie-infected mouse model in which PrP^Sc^ was identified in late-endosome-like organelles from brain homogenates, which were obtained by sequential centrifugation steps using a Nycodenz® density gradient. Analysis of these fractions by dot blot, western blot, and double-labeled immunogold electron microscopy identified the endosome-lysosome markers cathepsin B, mannose 6-phosphate receptor, ubiquitin-protein conjugates, and β-glucuronidase (Arnold et al., 1995). Because ILVs, the direct precursors of exosomes, are formed in late endosomes (Stoorvogel et al., 2002), the presence of PrP^Sc^ in these organelles may suggest a possible localization of PrP^Sc^ in exosomes.

Based on multiple analysis methods, including western blot, mass spectrometry, and morphological analysis, a later study found strong evidence supporting this possibility. PrP^c^ and PrP^Sc^ were found to be actively released into the extracellular space by PrP-expressing Rov cells before and after infection with sheep PrP^Sc^. Importantly, the study showed that exosomes containing PrP^Sc^ were infectious to other cells, suggesting a contribution of exosomes to the intercellular spread of prions *in-vivo* (Fevrier et al., 2004). Further support for this hypothesis came from studies reporting exosomal secretion of the endogenous prion protein by cultured primary rat cortical cells (Fauré et al., 2006), mouse hypothalamic neuronal GT1-7 cells (Vella et al., 2007), and mouse N2a cells (Alais et al., 2008; Veith et al., 2009). Additionally, exosomes derived from these cells were shown to introduce prions into uninfected recipient cells (Vella et al., 2007; Alais et al., 2008) and induce prion disease when inoculated in mice (Vella et al., 2007). The relationship between exosome release and intercellular prion transport was investigated also by Guo et al. who observed that stimulation of exosome release by treatment with the ionophore monensin (Savina et al., 2003) led to an increase in prion infectivity, whereas inhibition of exosome release using GW4869 (Guo et al., 2015) decreased prion transmission between rabbit kidney epithelial (RK13) and mouse GT1-7 cells (Guo et al., 2016).

In contrast to other neurodegenerative disorders, wherein only a small fraction of the offending proteins are released in exosomes, exosomes may be a major pathway for the spread of pathological proteoforms in prion diseases (Arellano-Anaya et al., 2015; Stuendl et al., 2016). Arellano-Anaya et al. (2015) showed that strains of PrP^sc^ from three different species were secreted into the culture medium of RK13 cells and were present in fractions containing exosomal markers and at typical densities of exosomes. Moreover, it was shown that these exosomal prion proteins retained their infectivity (Arellano-Anaya et al., 2015). Pan et al. observed that prion proteins were secreted in exosomes upon inhibition of cyclophillins by the immunosuppressive agent cyclosporine A, which usually leads to an accumulation of aggregated PrP^sc^ and its deposition in aggresomes in N2a and Chinese hamster ovary cells (Pan et al., 2018). The presented studies strongly indicate that different prion protein species are secreted via exosomes and therefore contribute, possibly to a high extent, to the spread of the misfolded, pathogenic protein in prion diseases.

## CNS-Derived Exosomes as a Source of Biomarkers for Neurodegenerative Diseases

Blood biomarkers are highly sought-after in the field of neurodegenerative diseases. They offer important advantages relative to expensive imaging modalities or the invasive lumbar puncture required for analysis of CSF biomarkers. However, drawbacks such as inconsistent results from different research groups and weak or non-existent correlation with disease severity or with CSF-derived or imaging biomarkers have hampered progress in this direction (Mehta and Adler, 2016; Lashley et al., 2018; Zhao et al., 2019). The relatively poor performance of blood-based biomarkers reflects the disconnect between the brain biochemistry and the blood composition, which is maintained by the BBB to protect the brain. As CNS-derived exosomes can cross the BBB into the blood and can be isolated from the blood, measurement of biomarkers in them offers an attractive solution for these issues.

The groups of Zhang at University of Washington, Seattle and Goetzl at University of California, San Francisco have pioneered this field establishing isolation protocols for neuronal exosomes, which were used as a novel source for neurodegenerative-disease biomarkers. Neuronal exosomes were obtained by immunoprecipitation using antibodies targeting the neuronal marker proteins NCAM or L1CAM (see section Isolation of CNS-Derived Blood Exosomes). NCAM is a neuronal cell adhesion protein that belongs to the immunoglobulin superfamily and is involved in cell-cell and cell-matrix interactions. L1CAM is an axonal glycoprotein that plays an important role in nervous-system development and its mutations cause neurological syndromes known as CRASH.

Using this methodology, Fiandaca et al. (2015) determined the levels of total tau, pT181-tau, pS396-tau and Aβ42 in neuronal exosomes in a cohort comprising patients with AD, patients with FTD, and matching cognitively normal control subjects. They found significantly higher levels of all four biomarkers in patients with AD and of pT181-tau and Aβ42 in patients with FTD compared to healthy controls. Impressively, their final model classified 96.4% of patients with AD and 87.5% of patients with FTD correctly and predicted the development of AD up to 10 years before the onset of clinical symptoms (Fiandaca et al., 2015).

Further analysis of neuronal exosomes obtained from the same cohort, demonstrated that levels of cathepsin D, LAMP-1, and ubiquitinated proteins, which are involved in the proteasomal and lysosomal degradation pathways, were significantly higher in patients with AD than in those with FTD. Similar to their initial study, the authors found that the concentration levels of the investigated proteins in neuronal exosomes from patients with AD were significantly distinct from those in age- and sex-matched healthy controls up to 10 years before the diagnosis (Goetzl et al., 2015). The results suggested that neuronal lysosomal dysfunction is an early event in the development of AD and may be useful as a predictive biomarker in prospective studies.

A following study by the Rissman group found that plasma-derived neuronal exosomal levels of pT181-tau, pS396-tau, and Aβ42 were increased, whereas the post-synaptic protein neurogranin and repressor element 1-silencing transcription factor (REST) levels were decreased in patients with AD or with mild cognitive impairment (MCI) converting to AD compared to normal subjects and patients with stable MCI that did not convert to AD (Winston et al., 2016). These promising results suggest that alterations of these neuronal-exosomal biomarkers could predict the conversion from MCI to AD.

An adaptation of the original procedure for isolation of neuronal exosomes allowed Goetzl et al. to enrich astrocyte-derived exosomes from plasma and subsequent analysis of biomarkers in these exosomes (Goetzl et al., 2016). Astrocyte-derived exosomes from patients with AD, patients with FTD, and healthy controls showed up to 20-fold higher levels of β-site amyloid β-protein precursor-cleaving enzyme 1, γ-secretase, Aβ42, soluble APPα and APPβ, glial-derived neurotrophic factor, pT181-tau, and pS396-tau compared to the concentrations measured in neuronal exosomes. Moreover, concentration levels of Aβ42 in astrocytic exosomes were lower in AD samples compared to the concentrations in healthy control samples, whereas pT181-tau, pS396-tau, and Aβ42 concentration in neuronal exosomes were significantly higher than in the control samples (Goetzl et al., 2016). In a recent study, Rissman's group showed that plasma-derived neuronal and astrocytic exosomes from patients with mild traumatic brain injury (mTBI) contained high levels of Aβ42 and low levels of neurogranin compared to healthy individuals with no history of TBI, suggesting that injury-associated proteins in these exosomes could be used as biomarkers for mTBI (Winston et al., 2019).

The Zhang group analyzed α-synuclein in neuronal exosomes from a large cohort of 267 patients with PD and 215 healthy controls and found that α-synuclein concentrations in the isolated exosomes were higher in the PD group compared to the control group. Although the diagnostic performance of neuronal exosomal α-synuclein was moderate (receiver operating characteristic (ROC) analysis AUC = 0.654, sensitivity = 70.1%, specificity = 52.9%), a significant cross-sectional correlation of neuronal exosomal α-synuclein was found with disease severity (Shi et al., 2014), suggesting that if a similar correlation were observed longitudinally, this biomarker could be useful for measuring PD progression and outcome measures of clinical trials. In a follow-up study, the same group demonstrated that tau protein levels in neuronal exosomes were elevated in patients with PD but not in patients with AD (Shi et al., 2016). In a longitudinal study, Wang et al. tested the utility of plasma α-synuclein and CNS-derived exosomal α-synuclein at baseline and in 2-year follow-up samples in a cohort comprising 256 individuals who might be at risk of PD. Their data showed that an increase in plasma α-synuclein at baseline and at follow-up could predict progression of cognitive decline in a subgroup of people with an increased PD risk, evidenced by hyposmia and reduced dopamine transporter imaging. In contrast, a decrease of α-synuclein in exosomes was associated with worsening of cognitive performance (Wang et al., 2018).

Recently, the protocol developed by Goetzl et al. was used by another group to determine the levels of DJ-1 and α-synuclein in neuronal exosomes from 39 patients with PD and 40 healthy controls (Zhao et al., 2019). Both, DJ-1 and α-synuclein were significantly higher in neuronal exosomes from patients with PD than in those from healthy controls whereas no significant differences were observed in total plasma, in agreement with the previous study by Shi et al. (2014). As in the previous study, ROC analysis yielded only a moderate discrimination between patients with PD and healthy controls even when both biomarkers were combined (Zhao et al., 2019).

In another new study, Aβ42, total tau, and pT181-tau were analyzed in two cohorts consisting of patients with AD, patients with amnestic MCI (aMCI), and healthy controls (Jia et al., 2019). The study included a discovery stage cohort comprising 28 patients with AD, 25 patients with aMCI, and 29 healthy controls, and a larger validation cohort consisting of 73 patients with AD, 71 patients with aMCI, and 72 healthy controls. The authors compared the biomarker concentration levels in the neuronal exosomes to the concentration of the same biomarkers in the CSF of all subjects. The data in both the discovery and validation cohorts showed that all three assessed biomarkers—Aβ42, total tau, and pT181-tau in neuronal exosomes were highest in patients with AD, significantly lower in patients with aMCI, and lowest in healthy controls. Encouragingly, the level of each exosomal biomarker showed a strong correlation with the respective CSF biomarker, suggesting that the measurement of these biomarkers in the neuronal exosomes could replace CSF analysis (Jia et al., 2019). A comprehensive summary of the studies described in this section is shown in Table 2.

**Table 2:** Selected publications analyzing biomarkers in CNS-derived blood exosomes.

**Exosome isolation method**	**Validation methods**	**Study cohort**	**Analyzed biomarkers**	**Outcome**	**References**
Immunocapture using anti-L1CAM antibody-coated M-270 Dynabeads	TEM, Western blot	PD: 267 HC: 215	Neuronal exosomal α-synuclein	α-synuclein: PD↑, Correlation with disease severity	Shi et al., 2014
Exosome precipitation and immunocapture using biotinylated anti-NCAM or anti-L1CAM antibodies and streptavidin-agarose resin	NTA	AD: 57 AC: 57 FTD: 16 FTC: 16 AD (preclinical and after AD diagnosis): 24	Neuronal exosomal Aβ42, total tau, pT181-tau, pS396-tau	Aβ42, total-tau, pT181-tau, pS396-tau: AD↑ Aβ42, pT181-tau: FTD↑ Aβ42, pT181-tau, pS396-tau: Preclinical AD↑ compared to AC, AD↑ compared to preclinical AD and AC	Fiandaca et al., 2015
Exosome precipitation and immunocapture using biotinylated anti-L1CAM antibody and streptavidin-polyacrylamide resin	NTA	AD: 26 AC: 26 FTD: 16 FTC: 16 AD (preclinical and after AD diagnosis): 20	Neuronal exosomal Cathepsin D, LAMP-1, Ubiquitin, HSP-70	Cathepsin D, LAMP-1, ubiquitinylated proteins: AD↑ compared to AC and FTD HSP70: AD↓ compared to AC, FTD↓ compared to FTC and AD Cathepsin D: FTD↑ compared to FTC Cathepsin D, LAMP-1, ubiquitinylated proteins: preclinical AD↑, AD↑ compared to AC HSP70: preclinical AD↓, AD↓ compared to AC	Goetzl et al., 2015
Exosome precipitation and immunocapture using biotinylated anti-L1CAM antibody and streptavidin-polyacrylamide resin	TEM, NTA	AD: 10 MCI: 20 MCI to AD converter: 20 HC: 10	Neuronal exosomal Aβ42, pT181-tau, pS396-tau	Aβ42, pT181-tau, pS396-tau: AD↑, MCI to AD converter↑ both compared to MCI and HC NRGN, REST: AD↓, MCI to AD converter↓ both compared to MCI and HC	Winston et al., 2016
Exosome precipitation and immunocapture by biotinylated anti-GLAST or anti-L1CAM antibodies and streptavidin-agarose resin	NTA	AD: 12 AC: 10 FTD: 14 FTC: 10	Neuronal and astrocytic exosomal BACE-1, γ-secretase, sAPPα, sAPPβ, Septin-8, GDNF, Aβ42, pT181-tau, pS396-tau	BACE-1, sAPPβ: AD↑, FTD n.s. GDNF: AD/MCI↓, FTD n.s. BACE-1, γ-secretase, Aβ42, sAPPα, sAPPβ, GDNF, pT181-tau, pS396-tau: ADE of all groups↑ compared to NDE	Goetzl et al., 2016
Immunocapture using anti-L1CAM antibody-coated M-270 Dynabeads	TEM, Western Blot, NTA	PD: 91 AD: 106 HC: 106	Neuronal exosomal total tau	Total-tau: PD↑ compared to AD and HC Correlation to disease duration and CSF tau in PD	Shi et al., 2016
Immunocapture using anti-L1CAM antibody-coated M-270 Dynabeads	Not determined	Normosmia/ no DAT reduction: 80 Hyposmia/ no DAT reduction: 133 Hyposmia/ DAT reduction: 43	Total plasma and neuronal exosomal α-synuclein	Total-α-synuclein: Hyposmic/ DAT reduction↑ at baseline and longitudinally NDE α-synuclein: Hyposmic/ DAT reduction↓ longitudinally Correlation with cognitive function and DAT imaging	Wang et al., 2018
Exosome precipitation and immunocapture by biotinylated anti-L1CAM antibody and streptavidin-agarose resin	TEM	PD: 39 HC: 40	Neuronal exosomal DJ-1 and α-synuclein	DJ-1 and α-synuclein: PD↑ compared to HC	Zhao et al., 2019
Exosome precipitation and immunocapture by biotinylated anti-NCAM antibody and streptavidin-agarose resin	TEM, Western blot	Discovery stage: AD: 28 aMCI: 25 HC: 29 Validation stage: AD: 73 aMCI: 71 HC: 72	Neuronal exosomal Aβ42, total-tau, pT181 tau	Aβ42, total-tau, pT181-tau: aMCI↑ compared to HC, AD↑ compared to aMCI and HC Exosomal biomarker correlate with respective CSF biomarker	Jia et al., 2019
Exosome precipitation and immunocapture by biotinylated anti-L1CAM or anti-GLAST antibodies immobilized on streptavidin-coated magnetic beads	FACS	mTBI: 19 HC: 20	Neuronal and astrocytic exosomal Aβ40, Aβ42, NRGN, NfL, total tau, pT181-tau, pS396-tau	Aβ42: mTBI↑ NRGN: mTBI↓ Aβ40, total-tau, NfL, pT181-tau, pS396-tau: mTBI n.d. or n.s.	Winston et al., 2019

*↑, increased; ↓, decreased; AC, Alzheimer's disease control; AD: Alzheimer‘s disease; ADE, astrocyte-derived exosomes; aMCI, amnestic mild cognitive impairment; Aβ40, amyloid β-protein 1-40; Aβ42, amyloid β-protein 1-42; BACE-1, β-site amyloid precursor protein-cleaving enzyme 1; CSF, cerebrospinal fluid; DAT, dopamine transporter; FACS, fluorescence activated cell sorting; FTC, frontotemporal dementia control; FTD: frontotemporal dementia; GDNF, glial-derived neurotrophic factor; GLAST, glutamate aspartate transporter; HC, healthy control; HSP70, heat shock protein 70; L1CAM, L1-cell adhesion molecule; LAMP-1, lysosomal-associated membrane protein 1; MCI, mild cognitive impairment; MOG, myelin oligodendrocyte glycoprotein; mTBI, mild traumatic brain injury; n.d., not detectable; n.s., not significant; NCAM, neuronal cell adhesion molecule; NDE, neuron-derived exosomes; NfL, neurofilament light; NRGN, neurogranin; NTA, nanoparticles tracking analysis; PD, Parkinson‘s disease; pS396-tau, tau phosphorylated at S396; pT181-tau, tau phosphorylated at T181; REST, repressor element 1-silencing transcription factor; sAPPα/β, soluble amyloid precursor protein α/β; TEM, transmission electron microscopy; TRPS, tunable resistive pulse sensing*.

These studies demonstrate the potential of CNS-derived blood exosomes as a source of diagnostic, prognostic, and progression biomarkers for neurodegenerative diseases. In addition to blood products, exosomes obtained from other biofluids, such as CSF and saliva, also have been used for diagnostic purposes (Yoo et al., 2018; Cao et al., 2019). Obtaining saliva from patients is less invasive than obtaining blood and saliva is easier to process because it does not coagulate. However, as a source of biomarkers in neurodegenerative (and systemic) diseases, saliva has been studied much less than plasma or serum, likely because variability and risk of contamination in saliva compared to blood are higher (Han et al., 2018; Cao et al., 2019). In contrast, the fidelity of biomarkers measured in CNS-derived blood exosomes as representing biochemical changes in the CNS has been found to be similar to that of CSF biomarkers, suggesting that the same level of confidence can be achieved using a substantially less invasive procedure. This is important in particular for measurement of treatment efficacy in clinical trials where multiple tests often are required during the trial. Similar to CSF biomarker studies, the data also suggest that biomarker panels likely will provide better diagnostic or prognostic power than single biomarkers (Fiandaca et al., 2015; Goetzl et al., 2016; Shi et al., 2016; Winston et al., 2016). A particular advantage of biomarker analysis in CNS-derived blood exosomes compared to CSF is the ability to compare the biomarkers in exosomes originating in different cell types.

## Isolation of CNS-Derived Blood Exosomes

Numerous methods for isolation of exosomes have been reported, including protocols based on ultracentrifugation, filtration, precipitation, immuno-affinity capture, and microfluidics arrays (Contreras-Naranjo et al., 2017; Doyle and Wang, 2019; Zhang et al., 2019). However, only a minority of the reported methods can yield a sufficient number of exosomes when starting from a typical patient sample, e.g., 0.5 mL, if the goal is to isolate subsequently CNS-derived exosomes for biomarker analysis. Therefore, we focus here on isolation methods that have been used successfully for isolating CNS-derived blood exosomes followed by biomarker analysis. For more general reviews on exosome isolation techniques (see Li et al., 2017; Doyle and Wang, 2019).

The use of blood plasma as a source of exosomes requires the addition of EDTA or heparin to prevent clotting and subsequent separation of plasma by centrifugation (e.g., 15 min at 2,500 g) (Goetzl et al., 2015). To isolate exosomes from human plasma, thrombin needs to be added to the plasma samples prior to proceeding with the actual isolation. Alternatively, serum can be obtained by allowing the blood to clot for 15–20 min at room temperature before separating the serum by centrifugation and the serum then can be used directly for exosome isolation. Addition of protease and phosphatase inhibitors to the samples and maintaining the clotting time consistent for all the samples under study is crucial for preventing degradation or modification of exosomal ingredients, especially those present in minute concentrations (Fiandaca et al., 2015; Goetzl et al., 2015). Additional precautionary measures should be taken for measurement of α-synuclein in CNS-derived exosomes isolated from the blood as minute quantities of erythocytic α-synuclein released upon hemolysis can contaminate the sample.

**Figure 3:**
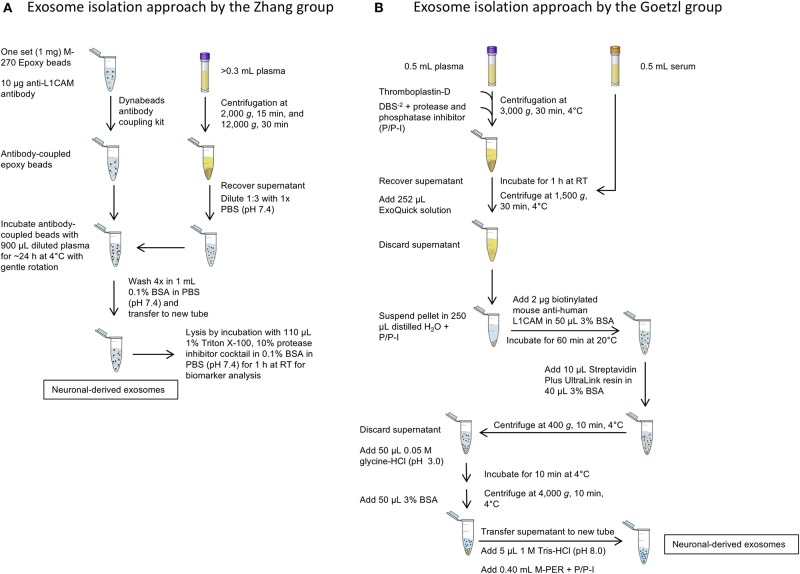
Isolation of CNS-derived exosomes from blood. **(A)** The protocol of the Zhang group relies on anti-L1CAM antibody-coupled epoxy beads, which are incubated directly with diluted plasma to bind neuronal exosomes. The following washing steps in 0.1% BSA remove unbound, non-neuronal exosomes in the sample. **(B)** The method described by Goetzl et al. The protocol uses first an exosome precipitation step by ExoQuick followed by capturing specifically neuronal exosomes with biotinylated anti-L1CAM antibodies and a streptavidin-conjugated resin. Subsequent washing steps remove non-neuronal exosomes as well as the antibody and resin to yield neuronal exosomes.

A side-by-side comparison of the protocols developed by the two first groups pioneering this field, the Zhang and Goetzl groups, is shown in Figure 3. The Zhang group was the first to describe a method for isolating CNS-derived exosomes from mouse and human plasma. Their approach used anti-L1CAM antibodies immobilized on superparamagnetic microbeads for immuno-capture of CNS-derived exosomes directly from plasma diluted 1:3 in phosphate-buffered saline without prior isolation of total exosomes (Figure 3A). They incubated diluted plasma samples with anti-L1CAM antibody-coated epoxy beads for 24 h with gentle rotation before proceeding to exosome release or lysis.

In the work of Goetzl and co-workers, the process included an initial polymer-assisted precipitation of extracellular vesicles from serum or plasma followed by immunoprecipitation using antibodies specific for NCAM or L1CAM to enrich CNS neuronal exosomes (Figure 3B; Fiandaca et al., 2015). They first incubated serum or thromboplastin-D-treated plasma samples with the non-specific PEG-based ExoQuick exosome precipitation solution to obtain a pellet of total exosomes, which was resuspended and the incubated with biotinylated anti-human NCAM or L1CAM antibodies to enrich neuronal exosomes by subsequent immunoprecipitation using a streptavidin-coated polyacrylamide resin (Goetzl et al., 2015). Similar approaches were used later by the groups of Kapogiannis, Rissman, and others (Winston et al., 2016; Doyle and Wang, 2019).

After capturing the exosomes by antibody-coated beads, according to both groups' protocols the beads are washed and the NCAM/L1CAM-positive exosomes can be eluted, e.g., for morphological analysis and for measurement of exosome number and size using methods, such as Nanoparticle Tracking Analysis (NTA), Tunable Resistive Pulse Sensing (TRPS) or Microfluidic Resistive Pulse Sensing (MRPS) (Shi et al., 2014; Goetzl et al., 2016; Winston et al., 2016; Jia et al., 2019; Yan et al., 2019). Alternatively, the exosomes may be lysed on the beads by treatment with different buffers, such as Mammalian Protein Extraction Reagent (M-PER) (Fiandaca et al., 2015; Goetzl et al., 2015) or other detergent-containing buffers for different downstream analyses and biomarker measurement (Shi et al., 2014, 2016; Wang et al., 2018). The methods used in different studies and the measured candidate biomarkers in CNS-derived blood exosomes for different neurological diseases and conditions are summarized in Table 2. The reported methods for isolation of CNS-derived exosomes from blood samples share wide similarities and differ mainly in the type of solid support used to immobilize the respective antibodies in the immunoprecipitation step. Although to date no study has compared side-by-side the different approaches, the efficiency and specificity of each method likely depends mainly on the specificity of the used antibody.

Adaptations of the protocol of Goetzl et al. have subsequently been applied for the isolation of astrocyte-derived blood exosomes using an anti-GLutamate ASpartate Transporter (GLAST) antibody (Goetzl et al., 2016, 2018). Our group was the first to successfully isolate oligodendrocyte-derived blood exosomes using anti-myelin oligodendrocyte glycoprotein (MOG) antibody coupled to magnetic Dynabeads (Dutta et al., 2018). A challenge in such studies is to confirm that the isolated exosomes indeed originated in the cell type intended. Such confirmation can be achieved using dot blots or western blots using antibodies against specific protein markers of the cells of origin. For example, enolase 2 or glutamate ionotropic receptor AMPA type subunit 1 (GRIA1) may be used to identify neuronal exosomes, glial fibrillary acidic protein (GFAP) for astrocytic exosomes, and myelin proteolipid protein for oligodendroglial exosomes. The challenge in such experiments is due to the low abundance of exosomes released specifically from these cell types into the blood (Doyle and Wang, 2019), which in our experience is ≤ 1% of the total serum/plasma exosomes. To address this challenge one must either isolate the exosomes starting with relatively large starting volumes of serum/plasma or use detection methods with higher sensitivity than those of dot blots or western blots.

It is also important to note that immunoprecipitation using NCAM or L1CAM does not provide exclusively CNS neuronal exosomes. NCAM and L1CAM are enriched in, but are not restricted to, neurons. They also may be present in microvesicles other than exosomes though no information exists currently regarding this possibility. According to the human protein atlas (https://www.proteinatlas.org), L1CAM is expressed mainly in the CNS, peripheral nervous system (PNS), and in distal renal tubules whereas NCAM is mainly observed in the CNS, PNS, adrenal gland, heart, and peptic cells. Proteomic analysis of L1CAM-captured exosomes from plasma showed higher concentrations of several CNS marker proteins, including phosphorylated tau, neuron-specific enolase, microtubule associated protein 2, neurofilament light chain (NfL), and L1CAM than in total exosome samples (i.e., before enrichment of neuronal exosomes) (Mustapic et al., 2017), suggesting that the majority of the exosomes indeed originated in CNS neurons. Nonetheless, the field should continue searching for, and testing, more specific markers that will allow isolation of purer exosome populations of CNS neurons and other cell types. Moreover, as different neurological diseases affect different brain regions, markers specific to a brain region or a neuron type, e.g., dopaminergic neurons for PD, may be developed in the future and allow biomarker analysis that would offer higher level of precision than general neuronal markers.

## Current Challenges and Future Perspectives

Extensive research in the last two decades has demonstrated that exosomes play a role in both physiological and pathological states of cells in the CNS. These vesicles function as intercellular communicators and serve as a vehicle for disposal of unwanted biological material. By carrying aggregated amyloidogenic proteins from cell to cell, exosomes contribute to the spread of these pathologic proteoforms in various neurodegenerative disorders. Impairment of the lysosomal and/or proteasomal pathways has been reported to increase disposal of pathogenic proteins via exosomes, contributing to disease spread in the CNS. However, the mechanisms involved in this process, the uptake of the released exosomes by specific recipient cells, the involvement of receptors and the impact of the lipid composition in the exosome membrane in these processes are yet to be elucidated.

Numerous studies have examined biofluid biomarkers for neurodegenerative diseases (Table 1). In AD, the most consistent biomarkers have been Aβ42, total tau, pT181-tau, and pS396-tau measured in CSF and more recently in neuronal exosomes (Tapiola et al., 2009; Fiandaca et al., 2015; Goetzl et al., 2016; Winston et al., 2016; Jia et al., 2019). The same biomarker panel has been shown to distinguish FTD from AD and healthy controls in multiple studies mostly using CSF as a biomarker source. In addition, although NfL is elevated in most neurodegenerative diseases, it was found to be significantly higher in FTD than in AD (Irwin et al., 2013; Fiandaca et al., 2015; Abu-Rumeileh et al., 2018; Goossens et al., 2018). DJ-1 and α-synuclein have been shown to be promising biomarkers in the diagnosis of PD. Results from studies involving neuronal exosomes have been demonstrated to be reproducible for α-synuclein (Shi et al., 2014; Dutta et al., 2018; Zhao et al., 2019) and for different forms of Aβ and tau (Fiandaca et al., 2015; Goetzl et al., 2016; Winston et al., 2016; Jia et al., 2019), and the acquired data in the neuronal exosomes correlated to those obtained from CSF samples (Jia et al., 2019). Increased CSF levels of tau and 14-3-3 proteins and decreased concentration levels of total PrP were found to be potential biomarkers for prion diseases (Otto et al., 1997; Llorens et al., 2018). NfL and phosphorylated forms of the neurofilament heavy chain have been shown consistently to be increased in the CSF, plasma, and serum of patients with ALS in several single- and multi-center studies (Boylan et al., 2013; Lehnert et al., 2014; Lu et al., 2015; Oeckl et al., 2016). However, NfL is associated with many neurodegenerative diseases and is likely to be more useful as an indicator of disease progression rather than diagnosis (Olsson et al., 2019; Preische et al., 2019). Therefore, TDP-43 measured in CSF may constitute a more promising biomarker for ALS as it was demonstrated to be a main component in the disease pathology (Neumann et al., 2006) and its concentration levels are elevated in patients with ALS compared to healthy controls (Kasai et al., 2009) and patients of other neurodegenerative or inflammatory diseases (Table 1; Noto et al., 2011; Hosokawa et al., 2014). Nevertheless, there is still a need for more specific biomarkers obtained through minimally invasive means for diagnosing patients reliably, ideally in early disease stages, monitor their disease progression, and evaluate clinical-trial outcomes. Similar needs exist for many rare neurodegenerative and neuromuscular diseases, many of which are genetic (e.g., ataxias, myotonic dystrophies) and can be diagnosed based on identifying the relevant mutant gene, but without reliable progression biomarkers, conducting successful clinical trials is a major challenge. Investigating new sources, such as CNS-derived exosomes, promises to generate robust biomarkers for neurodegenerative diseases through simple blood tests.

CNS-derived exosomes isolated from blood also have been shown to be a useful source of biomarkers for other neurological conditions, including stroke (Chen et al., 2016) and TBI (Winston et al., 2019), and for following brain processes that are not easily accessible, such as adult hippocampal neurogenesis (AHN) (Luarte et al., 2017). The enrichment of exosomes derived from specific brain cell types, such as neurons, astrocytes, and recently oligodendrocytes by immuno-capture likely will provide a more specific and useful source of diagnostic and progression biomarkers compared to plasma or serum themselves or total exosomes isolated from these biofluids. The fourth brain-cell type, microglia, presents a unique challenge because microglia share surface markers, which potentially could be used to distinguish their exosomes from the other brain-cell types, with peripheral monocytes and macrophages. In the absence of a unique microglial marker presented on the exosomal membrane, isolation of microglial exosomes from blood currently is not possible.

An important practical challenge in the methodology discussed above is the limited sample volume typically available from biobanks or providing clinics and the minute number of CNS-derived exosomes in such samples, necessitating the use of high-sensitivity detection methods, such as single molecule array (SIMOA, Quanterix, USA), electrochemiluminescence ELISA (Meso Scale Discovery, USA) or Immuno magnetic reduction (MagQu, Taiwan). Another potential difficulty, discussed more in personal communication than represented in the published literature, is the reproducibility of biomarker analysis in CNS-derived exosomes. The challenges are both the exosome-isolation process itself, which requires a high level of expertise and precision, and the characterization of the subsequent assays, which must be done for each assay separately using multiple independent exosome preparations for establishing acceptable intra- and inter-experiment coefficients of variation. There is still a need for reproducible, standardized protocols for isolation of exosomes and subsequent analysis of biomarkers of interest. Nonetheless, the examples discussed above demonstrate that exosome populations enriched from specific brain cell-types hold potential as a promising source of biomarkers for different neurodegenerative disorders and it will be particularly interesting to compare biomarkers in exosomes from different cell types side-by-side in the same samples. Finally, a major difficulty specific for development of diagnostic biomarkers for many neurodegenerative diseases is the absence of samples from patients with a validated diagnosis. To develop this field further, the establishment of biobanks containing pathologically validated samples from patients with neurodegenerative diseases and healthy controls is essential.

## Author Contributions

SH, SD, and GB conceived and wrote the manuscript.

### Conflict of Interest

The authors declare that the research was conducted in the absence of any commercial or financial relationships that could be construed as a potential conflict of interest.
